# Cohort Profile: The Adolescent and Young Adult Tracking Engagement and Management Skills (AYA TEAMS) longitudinal cohort of childhood cancer survivors in the United States

**DOI:** 10.1371/journal.pone.0361100

**Published:** 2026-11-02

**Authors:** Sara King-Dowling, Kelsey Woodard, Haley Faust, Sarah Drake, Lina Gov, Dava Szalda, Kemar V. Prussien, Jill P. Ginsberg, Wendy Hobbie, Carole A. Tucker, Lamia P. Barakat, Janet A. Deatrick, Yimei Li, Karen C. Burns, Kathryn Nielsen, Victor Flores, Nithya Ramaswamy, Margaret Jankowski, Bridget O’Hagan, Anneliese Wilkins, David R. Freyer, Ahna Pai, Lisa A. Schwartz

**Affiliations:** 1 Division of Oncology, Children’s Hospital of Philadelphia, Philadelphia, Pennsylvania, United States of America; 2 Division of Behavioral Medicine & Clinical Psychology, Patient & Family Wellness Center, Cancer and Blood Diseases Institute, Cincinnati Children’s Hospital Medical Center, Cincinnati, Ohio, United States of America; 3 Center for Biobehavioral Health, Nationwide Children’s Hospital Research Institute, Columbus, Ohio, United States of America; 4 Cancer and Blood Disease Institute, Children’s Hospital Los Angeles, Los Angeles, California, United States of America; 5 Perelman School of Medicine, University of Pennsylvania, Philadelphia, Pennsylvania, United States of America; 6 School of Health Professions, University of Texas - Medical Branch, Galveston, Texas, United States of America; 7 School of Nursing, University of Pennsylvania, Philadelphia, Pennsylvania, United States of America; 8 Department of Pediatrics, University of Cincinnati College of Medicine, Cincinnati, Ohio, United States of America; 9 Division of Oncology, Cancer and Blood Diseases Institute, Cincinnati Children’s Hospital Medical Center, Cincinnati, Ohio, United States of America; 10 USC Norris Comprehensive Cancer Center, Los Angeles, California, United States of America; 11 Department of Pediatrics, The Ohio State University College of Medicine, Columbus Ohio, United States of America; Tsinghua University, CHINA

## Abstract

**Purpose:**

To describe the rationale, methods, and baseline sample descriptives of the Adolescent and Young Adult Tracking Engagement and Management Skills (AYA TEAMS) cohort. The AYA TEAMS study is a longitudinal observational cohort study that aims to identify determinants and patterns of self-management and engagement in cancer-related long-term follow-up (LTFU) care and validate a novel transition readiness assessment among adolescent and young adult (AYA) survivors of childhood cancer.

**Participants:**

AYA survivors of childhood cancer (ages 16–25) and their caregivers were enrolled from 3 large pediatric oncology centers across the United States from 2020–2022 and followed for 2 years (minimum) to 3 years and 3 months (if transferred to adult care). AYA inclusion criteria were: past childhood cancer diagnosis (treated in a pediatric oncology setting), at least 2 years off-treatment, 5 years since diagnosis, engaged with the participating pediatric health care system (any department/specialty) within the last 18 months, cognitively able to complete study procedures, and English speaking. AYA completed a comprehensive battery of measures including assessments of self-management and transition readiness at baseline and annually for 2 years. For AYA transferred to adult care, separate measures were administered at the time of transfer (following last pediatric visit) and 15 months post transfer. Caregivers (English or Spanish-speaking) completed a single survey at baseline to capture family functioning, psychosocial risk, and transition readiness. Cancer diagnosis, treatment modalities, treatment-related late effects, and engagement in LTFU care were captured via electronic medical record review. In total, 709 AYA were enrolled and 587 were included in the final cohort [Mean age = 19.7 years, 52.5% female, 38.2% from racial and/or ethnic minoritized groups (REMG)]. The cohort was on average 7.3 years old at the time of diagnosis and 10.5 years off treatment. Half (52.5%) were survivors of leukemia/lymphoma, 38.0% solid tumors, and 9.5% central nervous system tumors. Three hundred and ninety-nine caregivers participated (90% mothers).

**Findings to date:**

Enrolled AYA excluded from the baseline cohort were more likely to be male, from REMG, and/or to enroll without a caregiver. Baseline cohort differences between study sites emerged for age, race and ethnicity, socioeconomic status, and treatment modalities and intensity.

**Future plans:**

Data collection was completed in April 2025. Findings from this cohort will elucidate important predictors of self-management and engagement in recommended annual LTFU and inform the design of interventions to reduce disengagement in LTFU.

## Introduction

Over 85% of children diagnosed with cancer under age 20 become long-term survivors, resulting in over 500,000 childhood cancer survivors in the United States [1–3]. As a consequence of their cancer treatment, approximately 70% of survivors of childhood cancer develop clinically significant late effects that can result in premature mortality, increased morbidity, and reduced quality of life [4–8]. Late effects often emerge during young adulthood and vary widely based on diagnosis and the types and doses of treatments received [4]. They can include subsequent malignant neoplasms, chronic health conditions (e.g., cardiovascular and lung disease, renal dysfunction, musculoskeletal problems, endocrinopathies), psychosocial difficulties (e.g., posttraumatic stress, social impairment), and/or cognitive deficits, and require ongoing surveillance and management [9].

Given the long-term late effects of treatment, it is critical that survivors of cancer demonstrate optimal self-management [10]. Self-management is the interaction of health behaviors and related processes that patients and families engage in to care for a chronic condition [11]. During adolescence and young adulthood, self-management is increasingly important as they begin to form and consolidate critical health behaviors and assume increasing responsibility for their own health independent of parental influence [12]. For adolescent and young adult (AYA) survivors of childhood cancer, self-management encompasses knowledge of cancer history and health risks, skills and confidence related to organization, planning, and/or administration of medical tasks, and engagement in cancer-related LTFU [13–15]. Concerningly, AYA self-management is generally insufficient as evidenced by disease management skills, knowledge, health promoting behaviors, and engagement in follow-up care that is less than optimal and exacerbates health vulnerabilities of AYA survivors [14,16–23].

Poor engagement in follow-up care is one well-established vulnerability for AYA survivors. The Institute of Medicine (now the National Academy of Medicine) and Children’s Oncology Group (COG) recommend annual cancer-focused long-term follow-up (LTFU) visits across the life span [5,24,25]. Such surveillance is needed to monitor for treatment-related late effects and adult malignancies that increase in incidence with age, and provide education for cancer prevention behaviors and psychosocial support, which may improve long-term survival [13,14,26]. Specialized pediatric and adult cancer survivorship centers are critical for providing this care, especially given primary care providers are often unfamiliar with the risks and needs of childhood cancer survivors [13–15]. Concerningly, LTFU attendance declines over time, particularly after the transition from pediatric to adult care, resulting in less than 30% of adult childhood cancer survivors receiving oncology LTFU [16,27–31]. In recent years, the NCI identified the combination of childhood cancer survivors facing higher risks for new cancers/morbidities and the decrease in LTFU care engagement as a health disparity and research priority [17].

Given the importance of life-long LTFU oncology care, it is also essential that AYA become ready to transition to, and engage in, the adult health care system once they age out of pediatric care [23]. Transition readiness is a gradual process, encompassing the purposeful, planned movement of AYA from pediatric- to adult-oriented health care [32]. It consists of multifactorial indicators that the AYA and those in their system of support (family, providers) can begin, continue, and finish the transition process [33,34]. As outlined in our Social-Ecological Model of AYA Readiness to Transition (SMART) [35], transition readiness is critical for AYA self-management in that it represents increasing maturity, knowledge, emotional readiness, and skills to sustain engagement in care. Increased transition readiness prior to transition is therefore expected to lead to higher engagement in pediatric-oriented care where transition planning will continue, indicating a potential transactional relationship between engagement in care and transition readiness [33,34]. However, advances in transition readiness research have been limited by historical reliance on cross-sectional data, lack of theory, and a paucity of valid measures of transition readiness, especially in pediatric oncology [35,36]. Additionally, few pediatric institutions offer standardized services for transferring childhood survivors to adult care [37–41], and only 8% of pediatric oncologists report using standardized transition readiness assessments to guide their care [42].

To better understand trajectories of AYA survivor self-management and transition readiness, we created the AYA Tracking Engagement and Management Skills (AYA TEAMS) longitudinal observational cohort consisting of AYA survivors of childhood cancer enrolled prior to the transition to adult care. Specifically, the AYA TEAMS study aims to: 1) validate a novel and comprehensive measure of transition readiness, the Transition Readiness Inventory (TRI); 2) test transactional relationships among transition readiness, self-management skills, and engagement in pediatric LTFU; and 3) identify predictors of engagement in adult LTFU care. Here we describe the cohort profile of the AYA TEAMS study, including design and methods, participant recruitment, and the demographic and clinical characteristics of the baseline sample across each of the 3 study sites. This cohort will serve as the basis from which we will test the aims of our longitudinal study in subsequent analysis and papers.

## Cohort description

### Study design

The AYA TEAMS study enrolled AYA survivors of childhood cancer from 3 large pediatric cancer centers across the United States: The Children’s Hospital of Philadelphia (CHOP, Northeast), Cincinnati Children’s Hospital Medical Center (CCHMC, Midwest), and Children’s Hospital Los Angeles (CHLA, West). Participants who were not transferred to adult care during the study completed three annual assessments (T1, T2, T3). If they were transferred between T1 and T3, they followed a modified schedule of assessments that included: 1) assessment after their last pediatric visit (designated transfer assessment #1, or Tr1) and 2) a 15-month follow-up (Tr15) to assess initial engagement in adult-focused LTFU after transfer. One parent or caregiver of each enrolled AYA was also invited to complete a single survey at baseline. A subset of AYA and caregivers from CHOP completed the TRI ~ 1 week (5–15 days) after baseline (T1) to evaluate test-retest reliability. See Fig 1 for study data collection timepoints. AYA completed online surveys at each timepoint, and medical history and attendance at LTFU care were abstracted from each patient’s electronic health record (EHR). All data were collected and managed using REDCap electronic data capture tools hosted at CHOP [43]. The CHOP IRB served as the central IRB and reviewed and approved this study protocol (IRB 17–013782).

**Fig 1:**
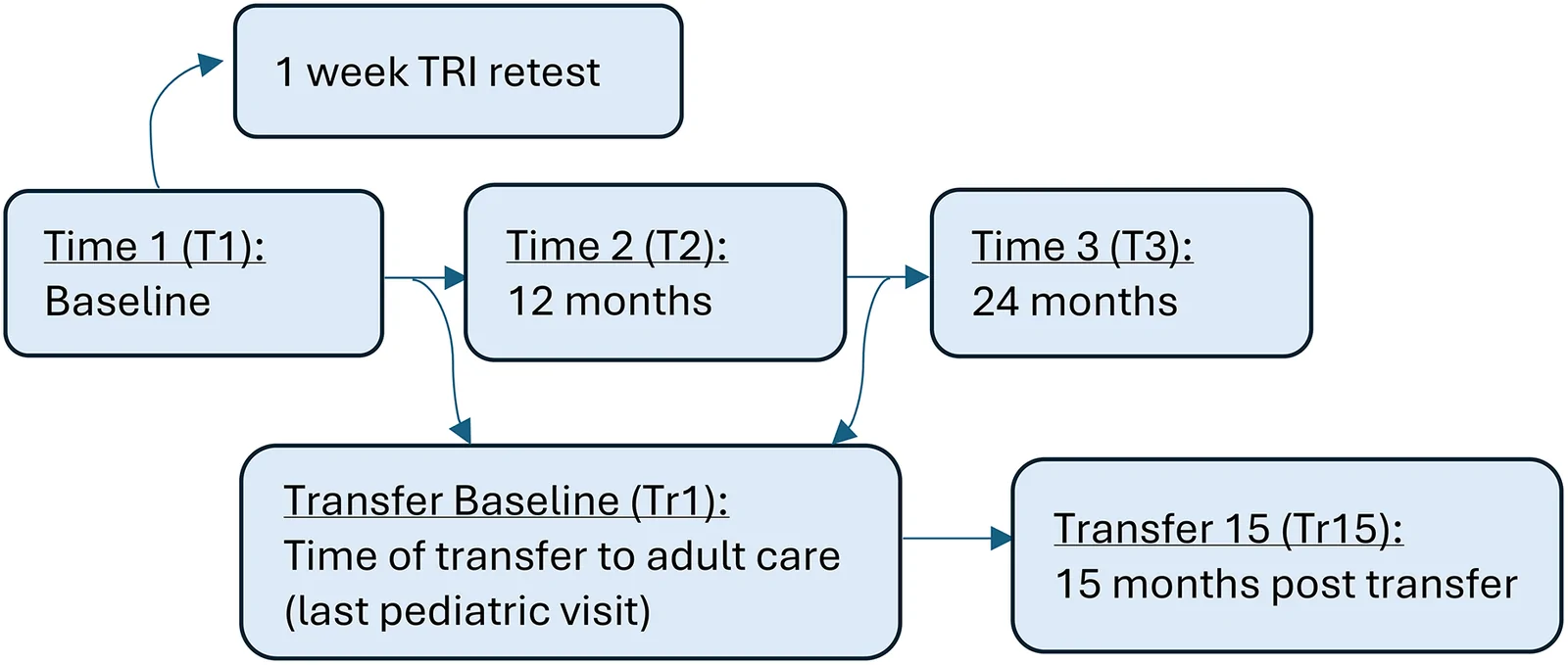
Data Collection Time Points. AYA= Adolescents and Young Adults; TRI=Transition Readiness Inventory. Note: Transfer to adult care had to occur prior to T3.

### Participant eligibility and recruitment

Inclusion criteria for AYA included: 1) 16–25 years old and a survivor of childhood cancer (treated in a pediatric oncology setting); 2) at least five years post diagnosis; 3) at least two years from end of treatment; 4) cognitively capable of completing study procedures; 5) ability to consent/assent and complete survey measures in English; 6) engaged within the enrolling pediatric hospital (any department/specialty) within the last 18 months; and 7) living in the U.S. Exclusion criteria included 1) already transferred to adult oncology care, 2) deceased or 3) provider did not provide approval to approach. Eligibility for caregivers included being a parent or primary caregiver of a non-married AYA participant and English or Spanish speaking. We were unable to include Spanish-only speaking AYA as many of the AYA survey measures were not available in Spanish. We attempted to enroll one parent/caregiver for each AYA enrolled, but AYA could elect to decline caregiver participation/contact. Eligible AYA were identified at the site level via EHR review and survivorship clinic lists and verified and approved by oncology clinicians. We aimed to enroll a demographically diverse sample by race/ethnicity and age and targeted recruitment efforts for underrepresented groups on an ongoing basis. AYA and caregivers were approached in clinic or via telephone. Caregivers and AYA over 18 years provided consent (in person or over the phone). AYA under 18 years provided assent and a parent or legal guardian provided consent, with AYA providing consent after they turned 18. AYA were passively withdrawn from the cohort and considered lost to follow-up if they completed less than half of the baseline (T1) survey.

### Sample size

The target sample size was determined based on the proposed longitudinal structural equation model (SEM) to test the predictors of, and transactional relationships between, transition readiness, self-management skills and engagement in LTFU. For SEM it is recommended to have at least 200 participants and 5–10 participants per parameter [44,45]. With 20–30 potential parameters, we estimated we would need ~300 AYA at T3 to adequately fit the SEM. Anticipating ~15% attrition at each timepoint and ~25% of AYA transferred to adult care before T3, we conservatively planned to enroll an evaluable sample of 600 AYA at T1 to end up with at least 300 AYA at T3.

### Study measures

Clinical characteristics including cancer diagnosis (type of cancer, age at diagnosis, relapse, second cancer), treatment history (including modalities and time off treatment), and late effects were collected via EHR review. Late effects, including current cancer-related health problems and future late effects risks related to treatment, were recorded based on documentation in the most recent oncology clinic note. Eighteen categories of late effects were abstracted: endocrine (including growth, thyroid and diabetes), cognitive, dermatological, vision, hearing, dental, cardiac, respiratory, kidney, gastrointestinal, reproductive, bladder/urinary, immunological, bone/joint, psychological, second cancers, neurological (including headaches, seizures and balance problems), and liver problems. If it was unclear whether a health problem was associated with past cancer and/or treatment history, it was reviewed with an oncologist to verify. All AYA were considered at least ‘at risk’ for psychological late effects. Intensity of Treatment Ratings (ITR) were rated using standardized procedures [46] from 1 (least intense) to 4 (most intense) based on diagnosis, stage/risk level, relapse, and number of treatment modalities received. All ratings were verified by an oncology clinician (survivorship physician or advanced practice provider), with discrepancies discussed and resolved.

Demographic characteristics (e.g., race, ethnicity, education) were self-reported at baseline by AYA and caregivers. AYA date of birth (to calculate exact age at enrollment), sex at birth, race, and ethnicity were also recorded from the EHR. Using participant addresses, neighborhood median income and low-income data were extracted from the U.S. Bureau of the Census 2020 Census Summary at the Census Tract (CT) level. A comprehensive battery of self-reported measures assessed transition readiness components, potential predictors of transition readiness, self-management skills, and engagement in LTFU (see Table 1). After the onset of COVID-19 and after ~15% of AYA had been enrolled, additional measures were added to assess COVID-19 exposures and impact (May 2020), risk and resiliency (June 2020) and COVID-19 vaccine hesitancy (December 2020) to use as potential covariates for planned analysis. REDCap survey links were distributed to participants via email and text message. See Fig 2 for the proposed transactional model among constructs.

**Table 1:** Self-reported survey measures.

Construct	Descriptive Measures	Time-point
Demographics	Race, ethnicity, age, education, household income, gender, sex, insurance status, marital status, employment status.	T1, CG
Health Behavior Engagement	Health behaviors: smoking, vaping, alcohol consumption, marijuana and other drug use, sun exposure, nutrition, screen time, physical activity, sleep (questions adapted from national health surveys).	T1, 2, 3, Tr1, Tr15
	**Convergent Validity Measures (Aim 1), informed by the SMART model**	
Developmental maturity	Psychosocial Maturity Inventory (PMI) [47] assesses developmental maturity across 3 subdomains of work, self-reliance and identity.	T1
Knowledge	Health Knowledge Inventory (HKI) [48] assesses knowledge of late effects and cancer history, by comparing self-report to EHR.	T1
Skills/Efficacy	The Perceived Health Competence Scale (PHCS) [49] assesses degree to which an individual feels capable of effectively managing his or her health outcomes.	T1
	Transition Readiness Assessment Questionnaire (TRAQ) [50] measures “readiness for change” in acquiring disease management and healthcare utilization skills related to transition to adult care.	T1
Beliefs/Expectations	Expectations Scale [51] measures patients’ perceptions of adult healthcare, developed for childhood cancer survivors.	T1
Relationships/Communication	Health Care Relationship Trust Scale (HCRT) revised [52] measures patient’s trust in healthcare providers, payers, and institutions.	T1
Psychosocial Functioning	Cancer Related Worries [51] measures survivors’ worries related to future risk and late effects.	T1
**Construct**	**Predictor Measures (socio-demographic, health-related, and family risk; Aim 2)**	
Perceptions of Health	The Health Competence Beliefs Inventory [53] assesses beliefs about health vulnerabilities, autonomy, cognitive competence, and satisfaction with health care.	T1
HRQoL/Health Status	PROMIS 25 (pediatric) & 29 (adult) Profile [54,55] assesses physical function/mobility, anxiety, depression, fatigue, sleep disturbance, social interactions, and pain interference and intensity.	T1
Cognitive Functioning	Childhood Cancer Survivor Study - Neurocognitive Questionnaire (CCSS-NCQ) – revised [56] assesses areas of neurocognitive functioning (e.g., working memory and executive functioning) most commonly affected by cancer treatment.	T1
Family Functioning	Family Assessment Device - General Functioning Subscale [57] assesses family cohesion and interaction.	T1
Family Life Difficulty	Family Life Difficulty subscale of the Family Management Measure [58] assesses the level of life difficulty attributed to condition management of the patient/child.	CG
Family Psychosocial Risk	Psychosocial Assessment Tool (PAT) measures family psychosocial/socio-demographic risk factors; developed in pediatric oncology [59].	CG
	**Self-Management Indices (Aims 2 and 3; TRI also Aim 1)**	
Engagement in Follow-up Care	Follow-up Attendance—Engagement in follow-up care (scheduling and attending) was determined by EHR review and supplemented by self-report (for patients transferred outside the hospital system).	T1, 2, 3, Tr1, Tr15
Self-management Skills Scale	Self-Management Skills Scale [51] assesses the cancer survivor’s level of agreement with regards to their ability to manage their health.	T1, 2, 3, Tr1,Tr15
Transition Readiness	Transition Readiness Inventory (TRI) assesses readiness to engage in adult-oriented care, with domains based on the SMART model. TRAQ—Convergent validity measure, also included as a legacy transition readiness measure.[50]	CG, T1, 2, 3, Tr1, Tr15
	**Patient Experience at Last Pediatric Appointment (for those transferred; Aim 3)**	
Perceived Transition Preparation	Adolescent Assessment of Preparation to Transition (ADAPT) [60] measures the quality of preparation for transition provided to the patient.	Tr1
Perceived Care of Chronic Condition	Patient Assessment of Chronic Illness Care (PACIC) [61] measures whether the patient receives patient centered care with an emphasis on self-management.	Tr1
	**COVID-19 -related measures**	
Exposure and Family Impact	COVID-19 Exposure and Family Impact Survey [62, 63] (CEFIS – AYA & CG (English/Spanish) – measures various aspects of the COVID epidemic that are likely to impact individuals and families and where COVID-19 may influence study outcomes.	CG; T1, 2, 3, Tr1, Tr15
Risk & Resilience	Risk/Resiliency Assessment [64] – understand the factors that increase risk for development of psychiatric disorders as well as promote resilience against disorders.	T1, 2, 3, Tr1, Tr15
COVID-19 vaccine	COVID-19 Vaccine hesitancy– thoughts on receiving vaccine and potential reasons for hesitancy.	CG; T1, 2, 3, Tr1,Tr15

AYA = Adolescent and young adult; CG = caregiver report; HRQoL = Health-related quality of life; PROMIS = Patient-Reported Outcomes Measurement Information System; SMART = Social-ecological Model of AYA Readiness to Transition; T1 = Time 1 (baseline); T2 = Time 2 (12 months); T3 = Time 3 (24 months); Tr1 = Time of transfer (last pediatric visit); Tr15 = 15 months post transfer.

**Fig 2:**
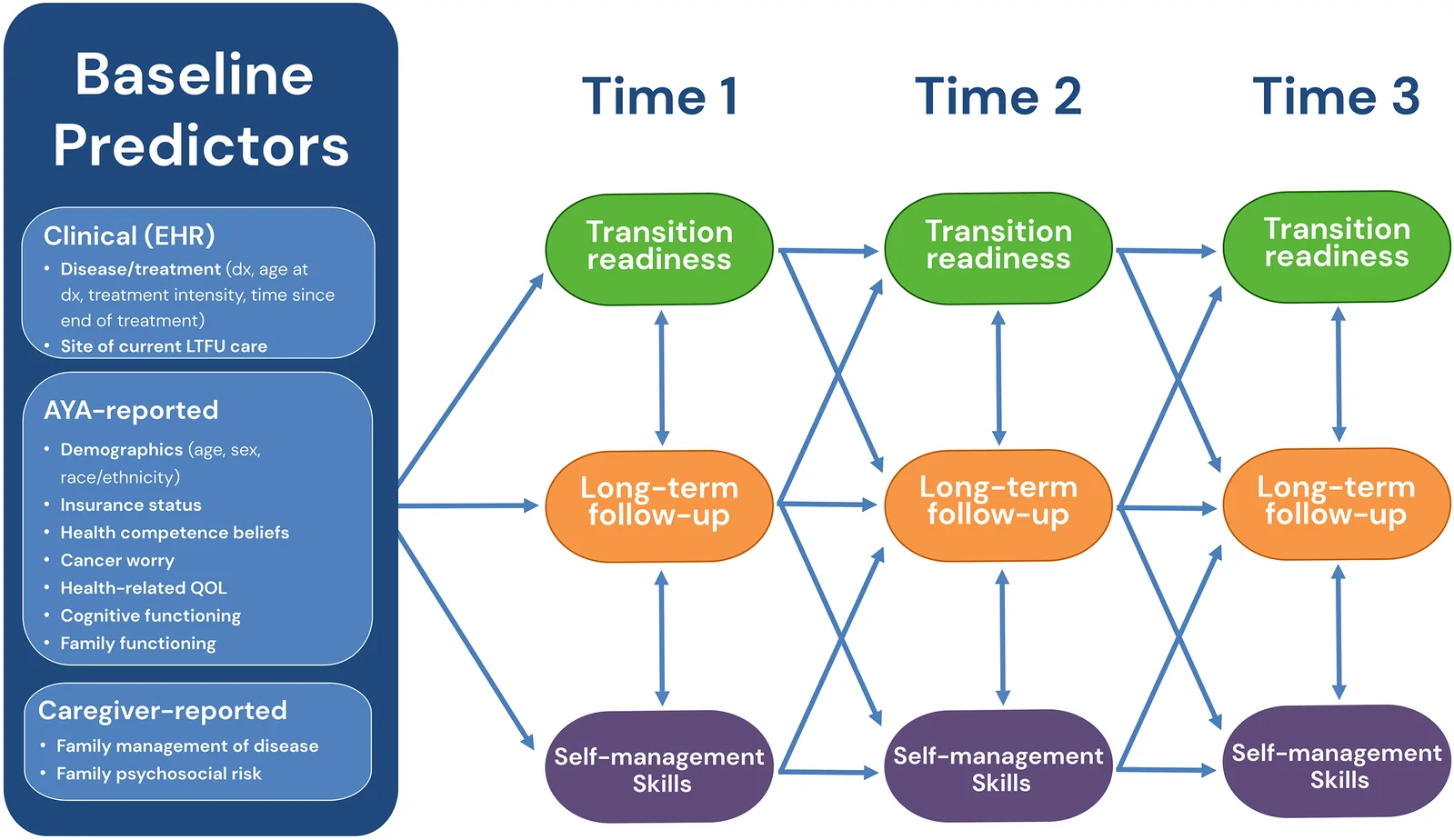
Proposed transactional model. AYA = Adolescent and young adult; Dx = diagnosis; EHR = Electronic Health Record; LTFU = long-term follow-up; QOL = quality of life.

### Statistical analysis

Descriptive statistics (means, SD, ranges, frequencies) for baseline sociodemographic and clinical characteristics were calculated to describe the AYA TEAMS baseline cohort. These results were examined for the sample in aggregate and by each study site. Differences in clinical and demographic characteristics between study sites were examined via Chi square, Fisher’s exact tests (for categorical variables with cell counts <5), and Analysis of Variance, with follow-up Bonferroni post-hoc adjustments for all significant site-level effects (significance level .05/3 = p < .017). Effect sizes for significant site main effects were also calculated: eta squared η^2^ for ANOVAS and Cramer’s V for contingency tables. We also conducted an exploratory analysis to examine differences between enrolled and eligible AYA included in the cohort and those who never completed baseline based on EHR-abstracted age, sex, race/ethnicity, and cancer diagnosis type, as well as caregiver enrollment and study site using Chi square and t-tests. All analyses were conducted in SPSS version 28.0.

### Participant recruitment

In total, 785 AYA were approached, 709 (90.3%) were enrolled between December 18, 2019 and August 2, 2022, 12 were withdrawn at baseline due to ineligibility, and of those eligible (n = 697) 587 (84.2%) participants completed at least half of the baseline (T1) measures survey and were included in the cohort (See Fig 3). For caregivers, of the 709 AYA enrolled, 518 (73.1%) caregivers were approached and of those 480 (92.7%) enrolled (See Fig 4). Across sites, the majority of AYA were recruited in person at clinic appointments (n = 413, 58.3%), with the remainder recruited remotely over the phone. For CHLA and CCHMC, phone-based recruitment was more prevalent (66% and 73%, respectively) largely due to COVID-19 pandemic restrictions (starting March 2020) and clinic preferences for limiting in-person patient contact. For phone-based recruitment we attempted to contact patients around the time of their oncology clinic visits.

**Fig 3:**
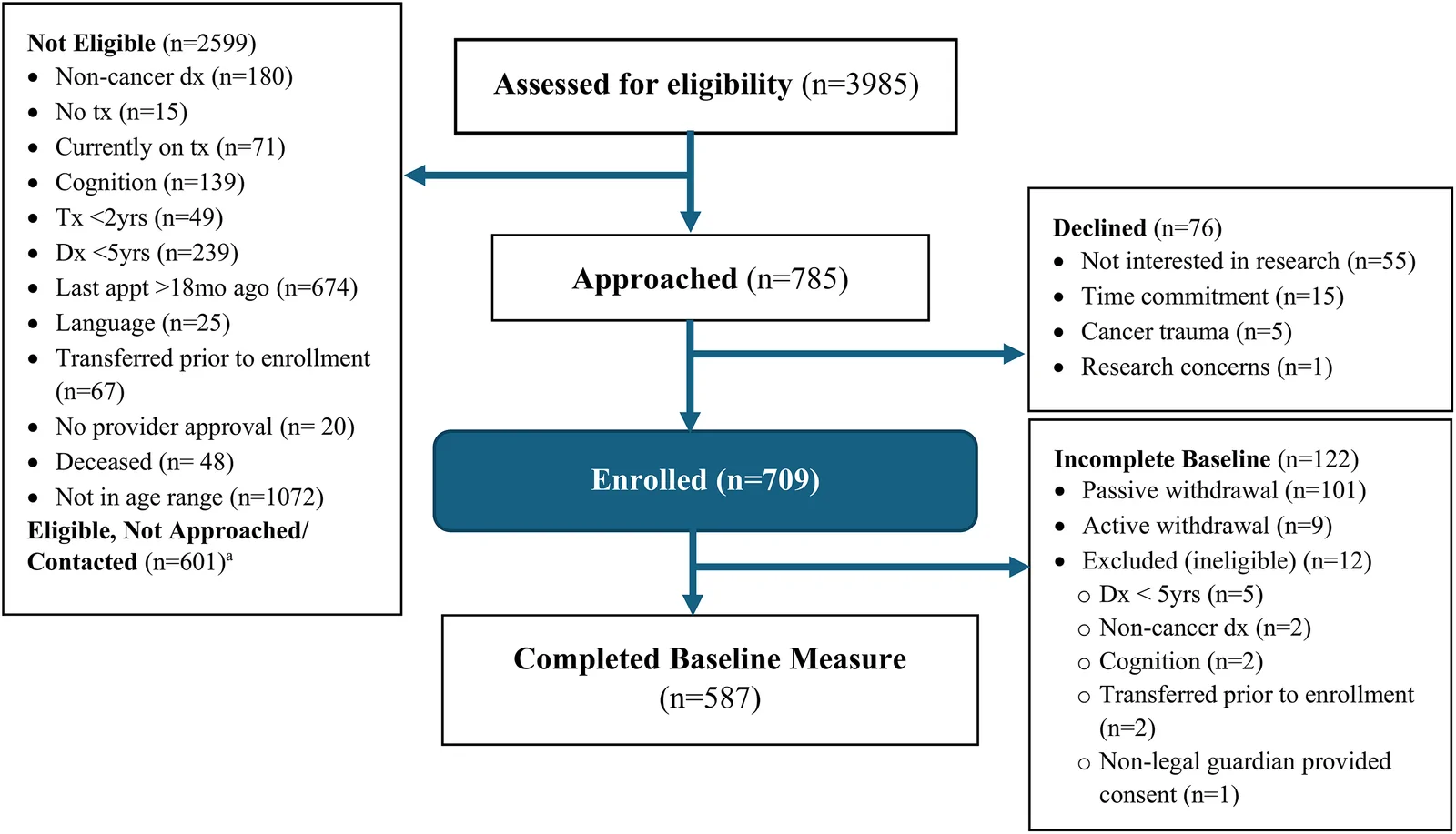
AYA participant flow diagram. dx=diagnosis; tx=treatment. ^a^Reasons for non-approach include: patients not attending clinic visits, unable to contact remotely, sites already meeting recruitment targets for demographic groups, overlapping recruitment with other studies.

**Fig 4:**
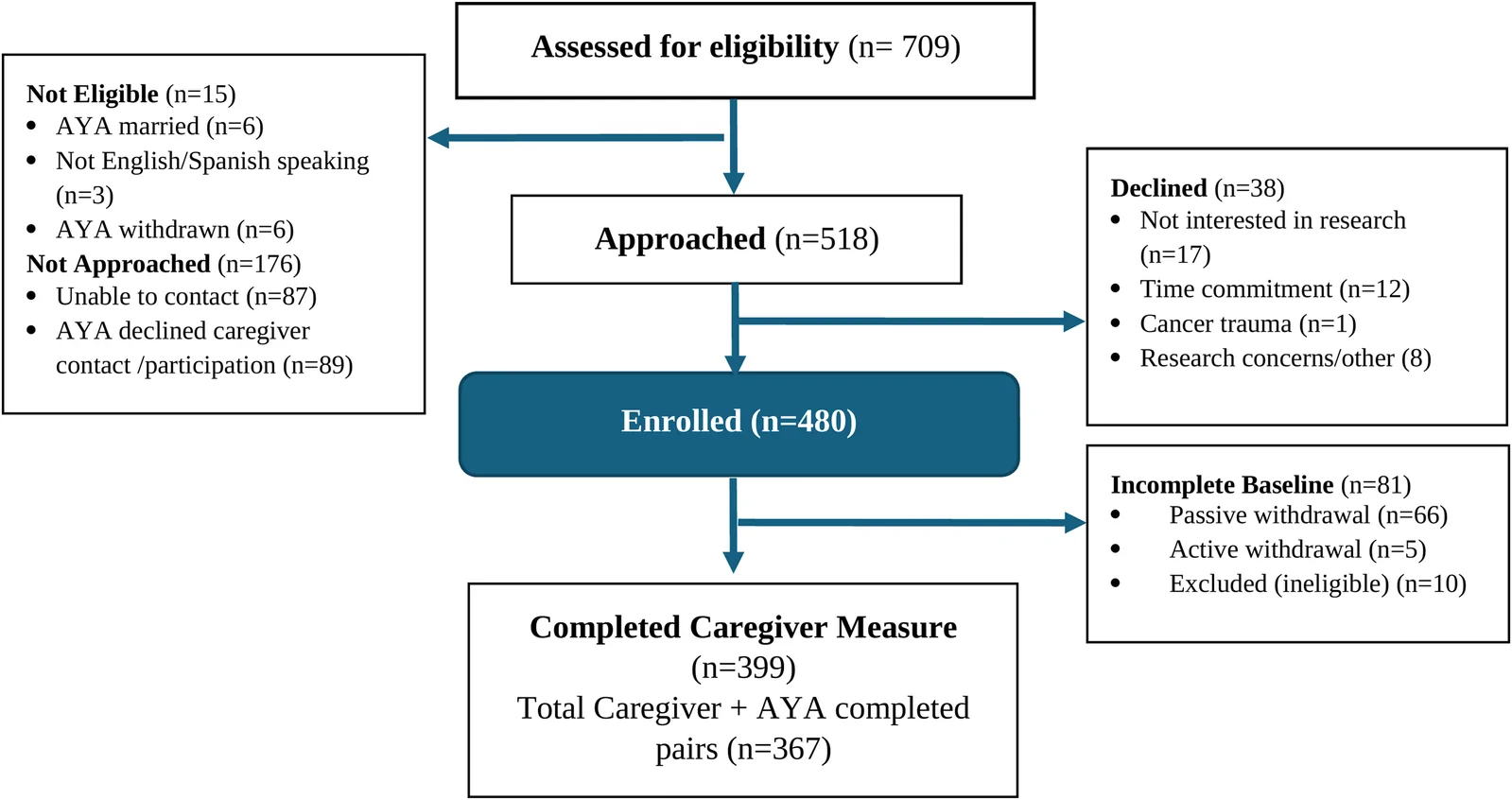
Caregiver participant flow diagram. AYA=adolescent/young adult.

### Participant characteristics

#### AYA.

Clinical and demographic characteristics of our baseline cohort at each site and combined can be found in Tables 2 and 3, respectively. The cohort was on average 7.3 years old at the time of diagnosis, 10.5 years off treatment, and about half the sample (53%) were survivors of leukemia or lymphoma. Almost all AYA had been previously seen in a dedicated cancer survivorship clinic (95%), with more than half (n = 336, 57.2%) enrolled on the same day of their oncology clinic visit, and 75.8% of the sample enrolled within 1 month. More than one-third of AYA self-reported being from a racial and/or ethnic minoritized group (REMG, 38%) and 52% identified as female gender.

**Table 2:** AYA clinical characteristics.

	CHOP n=300 (51.1%)	CHLA n = 137 (23.3%)	CCHMC n = 150 (25.6%)	Total N = 587
**Diagnosis Type n(%)**				
Leukemia/Lymphoma	153 (51.0)	77 (56.2)	78 (52.0)	308 (52.5)
Solid Tumor	121 (40.3)	47 (34.3)	55 (36.7)	223 (38.0)
Brain or CNS Tumor	26 (8.7)	13 (9.5)	17 (11.3)	56 (9.5)
**Cancer Relapse/Recurrence n(%)**	52 (17.3)	15 (10.9)	18 (12.0)	85 (14.5)
**Second Cancer n(%)** ^**+**^	14 (4.7)	3 (2.2)	4 (2.7)	21 (3.6)
**Age at Primary Diagnosis [M(SD), Range, Years]**	7.1 (5.0), 0.0-19.2	7.8 (4.8), 0.0-19.5	7.2 (4.9), 0.0-17.6	7.3 (4.9), 0.0-19.5
**Years off Treatment**^**d**^ **[M(SD), Range]**	10.4 (4.5), 2.6-23.6	9.6 (4.4), 2.0-20.0^c^	11.3 (5.0), 2.5-24.3^b^	10.5 (4.6), 2.0-24.3
Treatment Modalities n(%)*				
Surgery	132 (44.0)	57 (41.6)	59 (39.3)	248 (42.2)
Chemotherapy	289 (96.3)^c^	125 (91.2)	136 (90.7)^a^	550 (93.7)
Radiation	148 (49.3)^b^	40 (29.2)^a^	64 (42.7)	252 (42.9)
Bone Marrow Transplant	75 (25.0)^b,c^	8 (5.8)^a^	18 (12.0)^a^	101 (17.2)
Immunotherapy^e+^	20 (6.7)	4 (2.9)	3 (2.0)	27 (4.6)
**Intensity of Treatment Rating n(%)** ^**+**^				
1 (Least intense)	1 (0.3)^b,c^	7 (5.1)^a^	7 (4.7)^a^	15 (2.6)
2	90 (30.0)^b^	58 (42.3)^a^	54 (36.0)	202 (34.4)
3	120 (40.0)	54 (39.4)	61 (40.7)	235 (40.0)
4 (Most intense)	89 (29.7)^b,c^	18 (13.1)^a^	28 (18.7)^a^	135 (23.0)
**Number of Documented Current Late Effects [M(SD), range]**	2.6 (2.4)^c^, 0-11	2.2 (1.8), 0-9	1.8 (2.6)^a^, 0-6	2.3 (2.1), 0-11
**Number of Documented Future Late Effects Risks [M(SD), range]**	3.5 (2.0)^b,c^, 0-9	9.4 (2.6)^a,c^, 0-15	4.9 (2.3)^a,b^, 0-10	5.2 (3.3), 0-15
**Survivorship clinic patient n(%)**	284 (94.7)	132 (96.4)	143 (95.3)	559 (95.2)
**Days since last oncology visit at enrollment [M(SD), Range]**	26.8 (113.0) ^c^, 0-973	33.8 (80.7) ^c^, 0-527	247.8 (400.1) ^a,b^, 0–3648	84.9 (240.5), 0-3648

CNS = central nervous system; CHOP = Children’s Hospital of Philadelphia; CHLA = Children’s Hospital Los Angeles; CCHMC = Cincinnati Children’s Hospital Medical Center

^a^sig different from CHOP, p < .017

^b^sig different from CHLA, p < .017

^c^sig different from CCHMC, p < .017

^d^Years since completion of treatment based on most recent diagnosis

^e^includes targeted antibodies, immunomodulators, and CAR-T

* Note: Statistical tests conducted for each category separately.

+ Fishers Exact tests conducted due to cell counts <5

**Table 3:** AYA demographic characteristics.

	CHOP (n=300)	CHLA (n=137)	CCHMC (n=150)	Total (n= 587)
**Age at enrollment [M(SD), Range, Years]**	19.6 (2.3)^c^, 16.0-25.9	19.2 (1.9)^c^, 16.0-25.9	20.5 (3.0)^a,b^, 16.1-25.0	19.7 (2.5), 16.0-25.9
**Sex at birth from EHR n(%)**				
Female	166 (55.3)	73 (53.3)	69 (46.0)	308 (52.5)
Male	134 (44.7)	64 (46.7)	81 (54.0)	279 (47.5)
**Gender n(%)^+^**				
Female	164 (54.5)	72 (52.6)	68 (44.7)	304 (51.8)
Male	133 (44.3)	64 (46.7)	80 (53.3)	277 (47.2)
Other^d^	3 (1.0)	1 (0.7)	2 (1.3)	6 (1.0)
Race n(%)*				
American Indian/Alaskan Native^+^	3 (1.0)	2 (1.5)	2 (1.3)	7 (1.2)
Asian	20 (6.7)	12 (8.8)	8 (5.3)	40 (6.8)
Black or African American	39 (13.0)^c^	8 (5.8)	7 (4.7)^a^	54 (9.2)
Native Hawaiian/Pacific Islander^+^	0 (0)	1 (0.7)	0 (0)	1 (0.2)
White	242 (80.7)^b,c^	51 (37.2)^a,c^	138 (92.0)^a,b^	431 (73.4)
Other	17 (5.7)^b^	64 (46.7)^a,c^	6 (4.0)^b^	87 (14.8)
Missing	0 (0)	4 (2.9)	0 (0)	4 (0.7)
**Ethnicity n(%)**				
Hispanic	28 (9.3)^b^	95 (69.3)^a,c^	8 (5.3)^b^	131 (22.3)
Non-Hispanic	272 (90.7)^b^	42 (30.7)^a,c^	142 (94.7)^b^	456 (77.7)
**Non-Hispanic White n(%)**	214 (71.3)^b,c^	22 (16.1)^a,c^	127 (84.7)^a,b^	363 (61.8)
**Racial and/or Ethnic Minoritized Group n(%)**	86 (28.7)^b,c^	115 (83.9)^a,c^	23 (15.3) ^a,b^	224 (38.2)
**Marital Status n(%)^+^**				
Single, never married	288 (96.0)	137 (100)^c^	138 (92.0)^b^	563 (95.9)
Married	1 (0.3)^c^	0 (0)^c^	7 (4.7)^a,b^	8 (1.4)
Living with partner	10 (3.3)	0 (0)	4 (2.7)	14 (2.4)
Divorced/Separated	1 (0.3)	0 (0)	1 (0.7)	2 (0.3)
**Insurance Coverage n(%)^+^**				
Yes	289 (96.3)	131 (95.6)	147 (98.0)	567 (96.6)
I don’t Know	11 (3.7)	5 (3.6)	2 (1.3)	18 (3.1)
No insurance	0 (0)	1 (0.7)	1 (0.7)	2 (0.3)
Insurance Provider*^#^				
Employer/School/Self-Pay	13 (4.5)	6 (4.6)	11 (7.5)	30 (5.3)
Spouse^+^	0 (0)	0 (0)	1 (0.7)	1 (0.2)
Parents	242 (83.7)^b^	59 (45.0)^a,c^	119 (81.0)^b^	420 (74.1)
Government (Medicaid/CHIP)	41 (14.2)^b^	55 (42.0)^a,c^	21 (14.3)^b^	117 (20.6)
Military/Veteran^+^	1 (0.3)	0 (0)	3 (2.0)	4 (0.7)
Other^+^	1 (0.3)	3 (2.2)	0 (0)	4 (0.7)
I Don’t Know	17 (5.9)	14 (10.7)^c^	5 (3.4)^b^	36 (6.3)
**Highest Level of Education n(%)^+^**				
Grade school/Some high school	105 (35.0)	40 (29.2)	48 (32.0)	193 (32.9)
Completed high school	101 (33.7)^b,c^	70 (51.1)^a,c^	34 (22.7)^a,b^	205 (34.9)
Some college/vocational/ training school	65 (21.7)	21 (15.3)	32 (21.3)	118 (20.1)
Associate’s Degree	4 (1.3)^c^	2 (1.5)	8 (5.3^)a^	14 (2.4)
College graduate	22 (7.3)^c^	4 (2.9)^c^	25 (16.7)^a,b^	51 (8.7)
Post-graduate education	3 (1.0)	0 (0)	3 (2.0)	6 (1.0)
Current School/ Employment Status n(%)*				
Part-time student	20 (6.7)	14 (10.2)	13 (8.7)	47 (8.0)
Full-time student	215 (71.7)	94 (68.6)	91 (60.7)	400 (68.1)
Working part-time	102 (34.0)^b^	27 (19.7)^a^	48 (32.0)	177 (30.2)
Working full-time	40 (13.3)	9 (6.6)^c^	33 (22.0)^b^	82 (14.0)
Unemployed	38 (12.7)	29 (21.2)	22 (14.7)	89 (15.2)
Full-time homemaker/family caregiver^+^	1 (0.3)	0 (0)	1 (0.7)	2 (0.3)
Other^+^	4 (1.3)	6 (4.4)	1 (0.7)	11 (1.9)
**Median Neighborhood Income^e^ [USD, M(SD)]**	97,521 (39,871)^b,c^	77,334 (39,519)^a^	79,414 (30,846)^a^	88,222 (38,856)
Missing n(%)	14 (4.7)	6 (4.4)	10 (6.7)	30 (5.1)
**Low-income neighborhood^e^ n(%)**	35 (12.2)^b^	59 (45.0)^a,c^	21 (15.0)^b^	115 (20.6)
Missing n(%)	14 (4.7)	6 (4.4)	10 (6.7)	30 (5.1)

CHOP = Children’s Hospital of Philadelphia; CHLA = Children’s Hospital Los Angeles; CCHMC = Cincinnati Children’s Hospital Medical Center. EHR = electronic health record; USD = U.S. Dollars.

^a^sig different from CHOP, p < .017

^b^sig different from CHLA, p < .017

^c^sig different from CCHMC, p < .017

^d^Other genders described as: agender (n = 1), androgyne (n = 1), female non-binary (n = 1), non-binary (n = 1), none (n = 1), missing (n = 1).

^e^Based on participant address and 2020 U.S. census

* Note: participants selected all that apply so percentages add up to >100%. Statistical tests conducted for each category separately.

+ Fisher’s exact test conducted due to cell counts <5.

# Includes only participants who reported “yes” to having insurance coverage.

#### Caregivers.

In total, 399 caregivers completed the caregiver survey. Most caregivers were mothers (90%) and completed the English survey (94%). See Table 4 for detailed caregiver demographics.

**Table 4:** Caregiver demographic characteristics.

	CHOP (n = 259)	CHLA (n = 69)	CCHMC (n = 71)	Total (n = 399)
**Age at survey [(M/SD), range]**	50.9 (6.0)^b^, 36-64	48.3 (7.3)^a^, 34-75	49.7 (5.4), 38-62	50.3 (6.3), 34-75
Missing n(%)	2 (0.8)	0 (0)	0 (0)	2 (0.5)
**Gender n(%)** ^**+**^				
Female	235 (90.7)	66 (95.7)	65 (91.5)	366 (91.7)
Male	24 (9.3)	3 (4.3)	6 (8.5)	33 (8.3)
**Relationship to AYA n(%)** ^**+**^				
Mother	232 (89.6)	63 (91.3)	64 (90.1)	359 (90.0)
Father	24 (9.3)	3 (4.3)	6 (8.5)	33 (8.3)
Grandparent	3 (1.2)	2 (2.9)	1 (1.4)	6 (1.5)
Other	0 (0)	1 (1.4)	0 (0)	1 (0.3)
Race n(%)*				
American Indian/Alaskan Native^+^	3 (1.2)	1 (1.5)	0 (0)	4 (1.0)
Asian^+^	14 (5.4)	4 (5.8)	3 (4.2)	21 (5.3)
Black or African American^+^	21 (8.1)	4 (5.8)	1 (1.4)	26 (6.5)
Native Hawaiian/Pacific Islander^+^	1 (0.4)	0 (0)	0 (0)	1 (0.3)
White	219 (84.6)^b,c^	33 (47.8)^a,c^	69 (97.2)^a,b^	321 (80.5)
Other^+^	10 (3.9)^b^	24 (34.8)^a,c^	0 (0)^b^	34 (8.5)
Missing	0 (0)	3 (4.3)	0 (0)	3 (0.8)
**Ethnicity n(%)** ^**+**^				
Hispanic	14 (5.4)^b^	46 (66.7)^a,c^	2 (2.8)^b^	62 (15.5)
Non-Hispanic	245 (94.6)^b^	23 (33.3)^a,c^	69 (97.2)^b^	337 (84.5)
Non-Hispanic White n(%)	207 (79.9)^b,c^	15 (21.7)^a,c^	65 (91.5)^a,b^	287 (71.9)
**Racial and/or Ethnic Minoritized Group n(%)**	52 (20.1)^b,c^	54 (78.3)^a,c^	6 (8.5)^a,b^	112 (28.7)
**Survey language n(%)** ^**+**^				
English	256 (98.8)^b^	49 (71.0)^a,c^	71 (100)^b^	376 (94.2)
Spanish	3 (1.2)^b^	20 (29.0)^a,c^	0 (0)^b^	23 (5.8)
**Annual Household income [USD, n(%)]** ^**d+**^				
Less than 25,000	7 (2.7)^b^	17 (24.6)^a,c^	4 (5.6)^b^	28 (7.1)
25,000-49,000	25 (9.7)^b^	22 (31.9)^a,c^	6 (8.5)^b^	53 (13.3)
50,000-99,000	72 (27.8)^b^	6 (8.7)^a,c^	25 (35.2)^b^	103 (25.8)
100,000 or more	146 (56.4)^b^	21 (30.4)^a^	35 (49.3)	202 (50.6)
Don’t know	7 (2.7)	3 (4.3)	1 (1.4)	11 (2.8)
Missing	2 (0.8)	0 (0)	0 (0)	2 (0.5)
**Marital Status n(%)** ^**+**^				
Single	17 (6.6)^b^	12 (17.4)^a^	4 (5.6)	33 (8.3)
Married/Partnered	198 (76.4)	48 (69.6)	58 (81.7)	304 (76.2)
Separated/Divorced	39 (15.1)	5 (7.2)	6 (8.5)	50 (12.5)
Widowed	4 (1.5)	4 (5.8)	2 (2.8)	10 (2.5)
Other	0 (0)	0 (0)	1 (1.4)	1 (0.3)
Missing	1 (0.4)	0 (0)	0 (0)	1 (0.3)
**Highest Education n(%)** ^**+**^				
Less than high school	2 (0.8)^b^	15 (21.7)^a,c^	2 (2.8)^b^	19 (4.8)
Finished high school/GED	30 (11.6)	13 (18.8)	10 (14.1)	53 (13.3)
Started college/trade school	30 (11.6)	13 (18.8)	8 (11.3)	51 (12.8)
Finished college/trade school	123 (47.5)^b^	20 (29.0)^a^	30 (42.3)	173 (43.4)
Started master’s or doctorate program	13 (5.0)	1 (1.4)	2 (2.8)	16 (4.0)
Finished master’s or doctorate program	60 (23.2)	7 (10.1)	18 (25.4)	85 (21.3)
Missing	1 (0.4)	0 (0)	1 (1.4)	2 (0.5)

CHOP = Children’s Hospital of Philadelphia; CHLA = Children’s Hospital Los Angeles; CCHMC = Cincinnati Children’s Hospital Medical Center. USD = U.S. Dollars.

a sig different from CHOP, p < .017

b sig different from CHLA, p < .017

c sig different from CCHMC, p < .017

d Based on caregiver self-report

*Note: participants selected all that apply so percentages add up to >100%. Statistical tests conducted for each category separately.

+ Fisher’s exact tests conducted due to cell counts <5

### Findings to date

#### Baseline attrition.

Eligible AYA who consented (n = 697), but did not complete at least half of the baseline survey and excluded from the cohort (n = 110, 15.8%) were more likely male (69.1% vs. 47.5%, χ² = 17.23, p < .001, Cramer’s V = .16), non-white and/or Hispanic or unknown/refused race/ethnicity as indicated in the EHR (50.9% vs 36.1%, χ² = 8.6, p = .003, Cramer’s V = .11), and more likely to enroll alone without a caregiver (44.5% vs. 30.2%, χ² = 8.76, p = .003, Cramer’s V = .11) compared to AYA who were included in the cohort (n = 587). Attrition differed by site (χ² = 19.6, p < .001, Cramer’s V = .17) with CHLA experiencing the highest rates of dropout prior to completing baseline (25.9%) compared to CHOP (12.0%) and CCHMC (12.3%). No differences in baseline attrition were identified based on age at enrollment (19.8 vs. 19.7 years, t = 0.35, p = .73) or diagnosis type (χ² = 1.14, p = .57).

#### Cohort clinical characteristics across sites.

Among AYA included in the cohort (n = 587), no site differences across cancer types (χ² = 2.2, p = .69), cancer relapse (χ² = 4.1, p = .13), second cancer occurrence (Fisher’s exact p = .41), age at diagnosis (F = 1.01, p = .37), prior survivorship clinic attendance (χ² = 0.59, p = .74), surgery (χ² = 0.9, p = .63), or immunotherapy (Fisher’s exact, p = .06) were found. Site differences in other modalities of treatment emerged for chemotherapy (χ² = 7.3, p = .03, Cramer’s V = .11), radiation (χ² = 15.6, p < .001, Cramer’s V = .16), and bone marrow transplant (χ² = 28.1, p < .001, Cramer’s V = .22), with CHOP having a higher percentage of patients receiving chemotherapy (compared to CCHMC), radiation (compared to CHLA), and bone marrow transplant (compared to both sites). Relatedly, intensity of treatment differed across sites (Fishers exact p < .001) with AYA recruited from CHOP having the highest intensity of treatment, with the fewest participants receiving a score of 1 (minimally intensive) and the highest percentage of participants with an ITR of 4 (most intensive). Site differences were also found for number of current late effects (F = 7.3, p < .001, η^2^ = .024) and future late effects risks (F = 328.0, p < .001, η^2^ = .529). AYA from CHOP had a greater number of current late effects compared to AYA from CCHMC, whereas CHLA had the greatest number of late effects risks documented in the EHR. Time since completing treatment (F = 4.7, p = .010, η^2^ = .016) and time since last clinic visit (F = 54.7, p < .001, η^2^ = .158) also varied across site, with AYA from CCHMC off treatment for longer compared to those recruited from CHLA and enrolled further out from their last clinic visit compared to CHOP and CHLA participants (See Table 2).

#### Cohort demographics across sites.

Significant differences in many demographic variables were observed across sites regarding age (F = 12.1, p < .001, η^2^ = .040), education (Fisher’s exact p < .001, Cramer’s V = .20), marital status (Fisher’s exact p < .001, Cramer’s V = .14), neighborhood median income, (F = 18.0, p < .001, η^2^ = .061), living in a low income neighborhood (χ² = 62.6, p < .001, Cramer’s V = .34), racial and ethnic diversity (χ² = 166.3, p < .001, Cramer’s V = .53), and type of insurance (parent’s insurance: χ² = 75.2, p < .001, Cramer’s V = .36; public insurance: χ² = 47.4, p < .001, Cramer’s V = .29). AYA at CCHMC were slightly older, had achieved a higher level of education, and were more likely to be married compared to AYA from CHOP and CHLA. CHLA and CCHMC participants were from neighborhoods with lower average median income than CHOP participants, with CHLA participants more likely living in low-income neighborhoods than AYA from both other sites. CHLA had the greatest percentage of AYA from REMG, were less likely to be covered under their parent’s health insurance, and most likely to have public insurance compared to AYA from CHOP and CCHMC. Most AYA reported having some type of health insurance (97%) and this did not differ across sites (Fisher’s exact, p = .25). There were no differences in AYA sex (χ² = 3.5, p = .17) or gender (Fisher’s exact p = .40) distribution across sites (See Table 3).

Demographic site differences in the caregiver sample reflected that of AYA for race/ethnicity (χ² = 107.8, p < .001, Cramer’s V = .52) and household income (Fisher’s exact, p < .001, Cramer’s V = .32), with caregivers from CHLA more likely to identify as from REMG and reported lower household income compared to the other two sites. Site differences in caregiver age (F = 5.4, p = .005, η^2^ = .027), marital status (Fisher’s exact, p = .008, Cramer’s V = .164), and education (Fisher’s exact, p < .001, Cramer’s V = .29) were also found. Caregivers at CHLA were younger, more likely to be single, and less likely to have graduated college compared to CHOP caregivers, and less likely to have finished high school compared to both CHOP and CCHMC caregivers (See Table 4). No site differences in caregiver gender (Fisher’s exact, p = .46) or relationship to AYA (Fisher’s exact, p = .29) were found.

## Discussion

The AYA TEAMS cohort is a medically and demographically diverse cohort of AYA survivors of childhood cancer, including AYA across all cancer types and treatment modalities. According to recent (2022) U.S. childhood cancer statistics for survivors under age 30, approximately 48% are female, 51% are survivors of leukemia or lymphoma, 31% are survivors of a solid tumor and 18% are survivors of CNS tumor [65]. The lower proportion of CNS tumor survivors in our cohort (9.5%) may reflect exclusion of some survivors because of treatment-related cognitive impairment. It is important to highlight that this baseline cohort largely represents a group of AYA currently engaged in pediatric survivorship care with health insurance and excludes AYA who are not English-speaking. Therefore, this cohort may not be representative of all AYA survivors, particularly those who may be the most at risk for poor self-management who are already disengaged from care. This selection bias, in addition to the higher rates of attrition of males and AYA from REMG, may further bias the sample as these are groups that are known to be at greater risk for disengagement from LTFU [66]. Consequently, predictors identified in this cohort may not be fully generalizable, and future studies designed to specifically enroll these high-risk populations are needed. Although we will be able to assess engagement in LTFU through the medical record for patients who don’t complete follow-up surveys, future longitudinal analyses of this cohort will need to consider the potential impact of differential attrition and may require statistical approaches to address missing data. Of note, self-reported unemployment for AYA was high in this sample (15%); however, this is confounded by the high percentage of full-time students who also reported being unemployed as well as the COVID-19 pandemic. For caregivers, we enrolled 480 caregivers out of the 709 AYA enrolled (68%). This sample is likely biased towards caregivers who are more involved in their AYA’s daily life and health care (i.e., caregivers who attend oncology appointments with their child).

There were significant site differences across both demographic and clinical factors that will be important to consider in future cohort analyses. Demographic differences were expected and were part of the impetus for recruiting from three different geographic regions of the U.S. (Northeast, Midwest and West). Los Angeles has a much larger Hispanic population than Philadelphia or Cincinnati and this site was selected to help us increase the ethnic diversity of the AYA TEAMS sample. CCHMC does not typically transition its patients to adult care and retains patients into adulthood; therefore, it was expected that CCHMC would have a slightly older population of AYA survivors engaged with its pediatric health system. With regard to engagement in LTFU, although most (75%) patients were enrolled within 1 month of a pediatric oncology appointment, CCHMC patients tended to be enrolled farther out from their oncology LTFU appointments (Median = ~5 months). This was likely due to more significant COVID-19 restrictions impacting in-person clinic recruitment and will be important to account for when assessing engagement in LTFU across the study. Differences in treatment-related characteristics and late effects may relate to differing clinical practices across sites (e.g., treatment regimens, patient referral and transition patterns, EHR reporting) [67]. For example, this study includes survivors who completed treatment anywhere from 2 to 24 years ago; therefore, clinical differences may relate to the availability of new treatments at each hospital. In addition, survivorship care and transition practices have changed and evolved differently across sites such as variability in referral patterns to primary care, age at which sites would refer to adult care (if at all), shared care models within oncology (e.g., neuro-oncology or transplant teams continuing to follow survivors), and variable standards and templates for documenting late effects in the EHR. These differences highlight the importance of multi-site transition research and will be important to consider when analyzing and interpreting results.

### Future plans

To our knowledge, the AYA TEAMS study is among the first prospective cohorts of AYA-only off-treatment survivors of childhood cancer in the U.S, recruited during the critical developmental period of emerging adulthood and prior to the transition to adult-oriented care. This large cohort is essential for the final validation of the comprehensive Transition Readiness Inventory and to elucidate longitudinal and transactional patterns of, and risk for, poor self-management and disengagement in LTFU. Results from this cohort study will aid in designing tailored and targeted future interventions to enhance AYA self-management, including promotion of sustained engagement in cancer survivorship care.

### Strengths and limitations

- This study is one of the first known prospective cohorts of AYA-only long-term survivors of childhood cancer in the United States recruited from pediatric cancer centers.
- This study achieved high enrollment and retention rates across a medically and demographically diverse sample.
- Informed by multiple theoretical self-management models, this study will be able to examine predictors and transactional relationships of AYA survivor self-management, including engagement in pediatric and adult cancer-related long-term follow-up care.
- Reliance on English-speaking AYA and those currently engaged with the health care system are limitations.
